# Dispersal mode and spatial extent influence distance-decay patterns in pond metacommunities

**DOI:** 10.1371/journal.pone.0203119

**Published:** 2018-08-28

**Authors:** Irene Tornero, Dani Boix, Simonetta Bagella, Carla Pinto-Cruz, Maria Carmela Caria, Anabela Belo, Ana Lumbreras, Jordi Sala, Jordi Compte, Stéphanie Gascón

**Affiliations:** 1 GRECO, Institute of Aquatic Ecology, University of Girona, Girona, Spain; 2 Department of Chemistry and Pharmacy, University of Sassari, Sassari, Italy; 3 Departamento de Biologia, Escola de Ciências e Tecnologia, ICAAM - Instituto Ciências Agrárias e Ambientais Mediterrânicas, Universidade de Évora, Évora, Portugal; 4 ICAAM - Instituto Ciências Agrárias e Ambientais Mediterrânicas, Universidade de Évora, Évora, Portugal; University of Waikato, NEW ZEALAND

## Abstract

Assuming that dispersal modes or abilities can explain the different responses of organisms to geographic or environmental distances, the distance-decay relationship is a useful tool to evaluate the relative role of local environmental structuring *versus* regional control in community composition. Based on continuing the current theoretical framework on metacommunity dynamics and based on the predictive effect of distance on community similarity, we proposed a new framework that includes the effect of spatial extent. In addition, we tested the validity of our proposal by studying the community similarity among three biotic groups with different dispersal modes (macrofaunal active and passive dispersers and plants) from two pond networks, where one network had a small spatial extent, and the other network had an extent that was 4 times larger. Both pond networks have similar environmental variability. Overall, we found that environmental distance had larger effects than geographical distances in both pond networks. Moreover, our results suggested that species sorting is the main type of metacommunity dynamics shaping all biotic groups when the spatial extent is larger. In contrast, when the spatial extent is smaller, the observed distance-decay patterns suggested that different biotic groups were mainly governed by different metacommunity dynamics. While the distance-decay patterns of active dispersers better fit the trend that was expected when mass effects govern a metacommunity, passive dispersers showed a pattern that was expected when species sorting prevails. Finally, in the case of plants, it is difficult to associate their distance-decay patterns with one type of metacommunity dynamics.

## Introduction

Metacommunity ecology describes a group of local communities that interact with each other through dispersal and generate both local and regional interactions that influence local community assemblages [1–3]. Thus, metacommunity dynamics affect regional biotas, and this effect feeds back into the patterns of local variation [4,5]. Recently, community ecology has been attempting to elucidate the specific role of regional and local processes in determining metacommunity functioning [6–9]. In this sense, assessing the relative importance of environmental control against spatial distances appears to be a crucial aspect to disentangle which type of metacommunity dynamics is acting [6].

The distance-decay of similarity is a valuable tool to understand species assemblage responses to environmental and spatial variability [10]. For instance, a decay in similarity is usually observed when geographical distance between patches increases due to the dispersal limitation encountered when some organisms disperse across the landscape, and hence, widely separated points will harbour different communities [11]. Similarly, environmental distance decay might be observed when communities are environmentally controlled [12]. In fact, it is possible that both distances affect metacommunity dynamics and structure, and measuring the distances is an approach to evaluating the relative role of the spatial configuration of patches versus patch environmental variability [13,14]. Interestingly, a proposal has been developed [12] that links the patterns observed in the distance-decay of similarity and the dynamics governing metacommunities. Based on that proposal, we can identify three predictions that differ in terms of the effect of distance on community similarity according to three metacommunity paradigms: 1) species sorting, when a decay in similarity is observed for the environmental distance but not for the geographic distance; 2) neutral model, which is the opposite situation to the previous one, showing a decay in similarity for the geographic distance but not for the environmental distance, and 3) mass effects, showing a significant decay in similarity for both distances. However, Heino’s proposal does not incorporate the effect of the spatial extent on the rate of decay (i.e., the slope) of community similarity, which is usually lower at larger scales [15].

On the other hand, to fully understand the importance of the spatial configuration of patches, it is essential to recognize that different organisms may have a different perception of the same landscape. Accordingly, distance-decay relationships should be different depending on the dispersal mode of the organisms because of their different landscape perception [15–17]. Thus, when considering geographical distance, more actively dispersing taxa should exhibit a smoother decrease in similarities because the organisms would be less affected by barriers and would look for adequate habitats over larger distances [17,18], whereas more passive dispersers would exhibit a greater decrease [13,17,19]. In addition, when considering environmental distances, active dispersers should be more related with an environmental distance than passive dispersers since the former present less dispersal limitations and can better track environmental changes [20]. However, dispersal ability not only determines organism landscape perceptions (i.e., connectivity thresholds) but also acts as a key trait when establishing possible environmental controls on organisms (e.g., [20–22]). All these findings confirm the great importance of organism dispersal ability in determining metacommunity functioning [23], since environmental control, which is a central issue in some of the existing metacommunity paradigms (i.e., species sorting; [4]), could be minimized due to, for example, high dispersal rates. In turn, these high dispersal rates could be the cause of mass effects, another well-known metacommunity dynamics [24]. Therefore, the development of simultaneous studies on several biotic groups (differentiated according to their dispersal abilities) and in the same localities might be a good mechanism for gauging the importance of dispersal against the strength of local conditions [15] and thus would help to better understand metacommunity functioning. Moreover, dispersal ability also likely determines the degree at which the spatial extent becomes too large to encompass a metacommunity [12], and this degree is essential to establishing the spatial extent of the study. For many organisms (with the possible exception of unicellular organisms ([25,26] but see [27,28]), the spatial scale that encompasses the metacommunity is likely to be relatively small, usually delimited by physical boundaries (e.g., a drainage basin for stream organisms) [12]. Surprisingly, many distance-decay studies have been conducted at a continental or inter-continental scale [29], with studies rarely considering smaller extents (i.e., under 10 km) [30].

**Fig 1:**
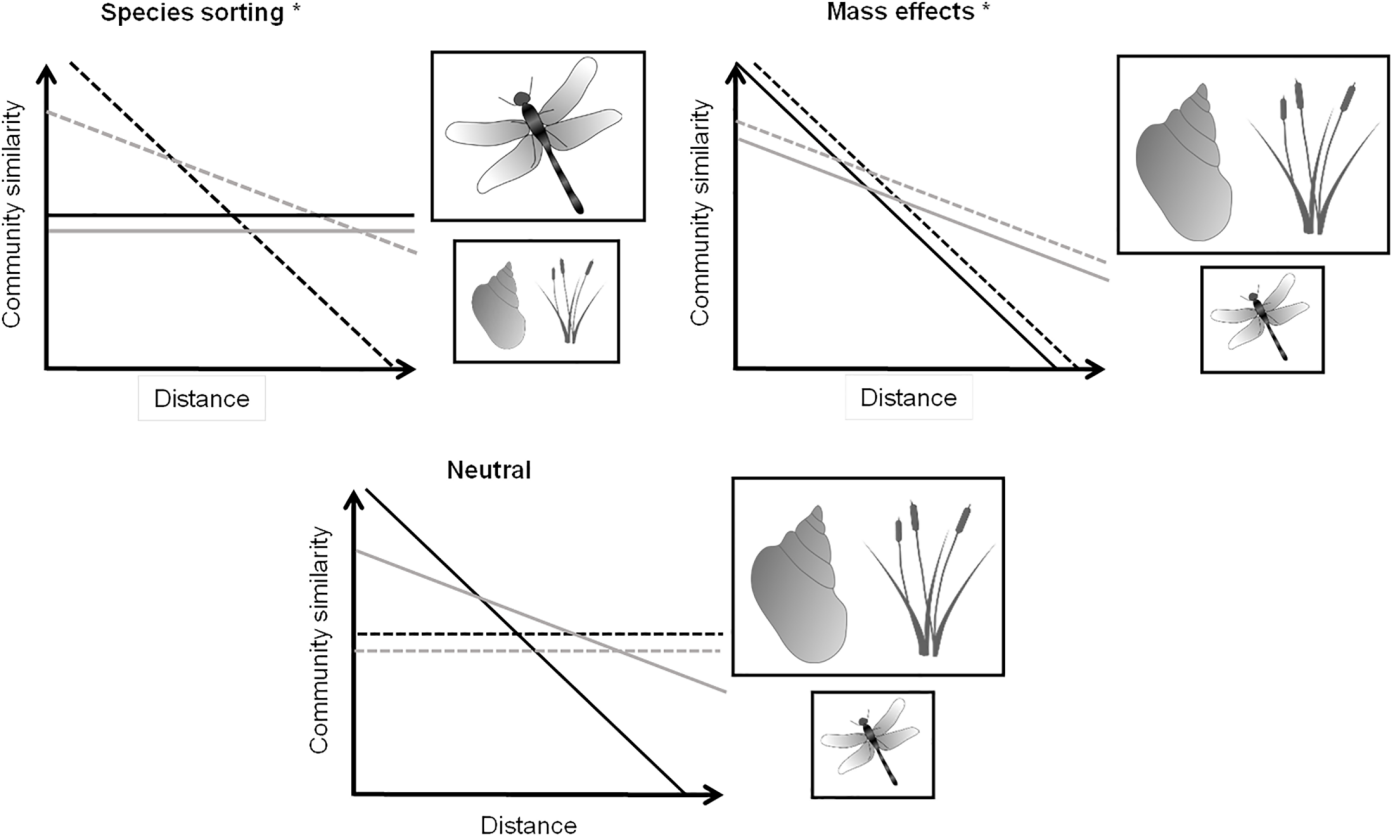
Conceptual scheme denoting the decrease in community similarity along geographic and environmental gradients taking into account the different types of metacommunity dynamics. The different sizes of organism symbols represent the higher (big symbol) or lower (small symbol) importance of the type of metacommunity dynamics for each biotic group. Grey lines indicate the response in the LEPN (large extent pond network) and black lines in the SEPN (small extent pond network). Solid lines indicate geographic distance, and dashed lines indicate environmental distance. The asterisk denotes the types of metacommunity dynamics that increase in importance at the surveyed small spatial extents [32,34]. The figure is modified from Heino [12]. Credits: Symbols courtesy of the Integration and Application Network, University of Maryland Center for Environmental Science (ian.umces.edu/symbols/).

Considering all the existing information, we propose a new framework that links distance-decay patterns to different metacommunity dynamics taking into account not only the dispersal ability of organisms but also the spatial extent of the study site (Fig 1). The aim of the present study is to test the validity of this framework with empirical data obtained from two sites with different spatial extent and to consider several biotic groups that differ in their dispersal abilities. To achieve this aim, we studied the community similarity of three biotic groups with contrasting dispersal strategies (macrofaunal active and passive dispersers and plants) in two pond networks with similar environmental variability (i.e., similar environmental gradients) but different spatial extent (one pond network was 4 times larger than the other pond network). The two pond networks have a relatively small spatial extent (below 10 km), and they are consistent with the natural size of the pond cluster, so the spatial extent of each pond network studied was delimited by an identified physical boundary. We tried to maximize the environmental variability (i.e., environmental gradient) within each study area, considering the broadest available range of pond sizes. Thus, following our framework, we first expected that, in the smaller extent pond network, the decay in community similarity would be steeper than that in the larger pond network [15,19,30] (see Fig 1). Second, we also expected that active dispersers would be more related to the environment (i.e., fitting under the expected trend for the species sorting paradigm) [31] than passive dispersers, and thus, we expected a stronger relationship with geographic distance of the latter [32,33]. Consequently, passive dispersers might show a distance-decay pattern closer to the pattern expected under mass effects or neutral model.

## Materials and methods

### Ethics statements

The field studies were conducted in two pond networks that are included under a Special Area of Conservation for the Natura 2000 Network (ITB041112-Giara di Gesturi; [35] and PTCON0012; [36]). The field studies were approved by the Ministerio de Economía y Competitividad of the Spanish Government, the Instituto da Conservação da Natureza e das Florestas of the Portuguese Government, the local Administration of Sardinia and the School of Doctoral Studies from the University of Girona. Thus, João Alves (as the director of the Departamento de Conservação da Natureza e Florestas do Algarve), Pedro Portela and Pedro Alverca from the PNSACV (Southwest Alentejo and Vicentine Coast Natural Park) in Portugal and the local authorities from the Giara Park in Sardinia permitted the sampling of ponds in each park area.

Some amphibian species are included in conservation international agreements, such as the Convention on the Conservation of European Wildlife and Natural Habitats. Consequently, adults of amphibians were in situ identified, measured, and immediately released. Although the sampling method followed (dip-net sweeps) was mainly designed for macroinvertebrate species and it was not planned to capture amphibians, some amphibian tadpoles were accidentally captured. However, this method does not allow us to see if we have captured tadpoles among the whole sample until we process the sample in the laboratory, therefore we only used the fixative (ethanol 96%) for the immediate conservation of the samples.

### Study sites

We selected two pond networks with similar environmental variability [37] but with different spatial extents: one pond network with a smaller extent (where the maximum distance between two ponds is 1.4 km) and the other pond network with a larger extent (where the maximum distance between two ponds is 5.3 km). Thus, the large extent pond network (hereafter LEPN) is almost 4 times larger than the small extent pond network (hereafter SEPN). For the present study, 11 temporary ponds, encompassing a wide range of sizes (from 245 to 78 652 m^2^ in SEPN and from 565 to 58 720 m^2^ in LEPN), were sampled in each pond network.

The SEPN is located in Vila Nova de Milfontes (SW Portugal) on a coastal, sandy plateau that ranges 50–150 m above sea level and is carved in Palaeozoic schist and covered by sandstone types—sands, sandstone and conglomerates [38]. The climate is Mediterranean with an oceanic influence, and the ponds fill mainly with rain water [38,39], although they are also possibly fed by groundwater [40]. The wet period usually lasts from November to March, and the dry period lasts from March to November, although there are both inter- and intra-annual variations. The flora and the fauna of these ponds have previously been studied (see [36,38,40–43]).

The LEPN is located in Giara di Gesturi (Sardinia, Italy) on a steep-sided basaltic plateau of 42 km^2^ in southern Sardinia on hydromorphic soils, with a clay texture and slow drainage. The climate is Mesomediterranean with a seasonal distribution of the rainfall, which is at a minimum in the summer and a maximum in the autumn. Snowfall on the plateau is not rare. All the ponds are filled by rain water and are temporary with a hydroperiod that usually lasts from October to June. The hydroperiod is followed by a dry period from June to October. Some information on the flora and the faunal groups of these ecosystems has been previously reported in several works (e.g., [44–49]).

### Data collection and processing

#### Environmental and geographic parameters

Water temperature (T), dissolved oxygen, conductivity, pH (model Hach HQ30d) and water-column depth were measured *in situ* during the sampling campaigns in LEPN and SEPN in April 2012 and 2013, respectively. Filtered water samples (250 mL) and unfiltered water samples (250 mL) were collected in each pond and frozen immediately. The dissolved inorganic nutrients (ammonia, nitrite, nitrate, phosphate) were measured from the filtered water samples with the ion chromatography system DIONEX ICS-5000. Dissolved Inorganic Nitrogen (DIN) was then calculated as the sum of the concentrations of ammonia, nitrite and nitrate. Total Inorganic Carbon (TIC), Dissolved Inorganic Carbon (DIC), Dissolved Organic Carbon (DOC), and Total Organic Carbon (TOC) were analysed using the TOC analyser Shimadzu TOC-V CSH and following UNE-EN 1484: 1998 guidelines. Total nutrients [total nitrogen (TN) and phosphorus (TP)] were analysed from unfiltered water samples, following Grasshoff *et al*. [50]. A nutrient limitation indicator was assessed using the ratio between DIN and TP (molar DIN/molar TP; [51]). Planktonic chlorophyll-*a* (Chla) content was extracted using 90% acetone, after filtering water samples (Whatman GF/F filters). Chlorophyll-*a* analyses were carried out with a high-pressure liquid chromatography (HPLC; Waters Pump 1500 Series with an autosampler injector (Waters 717 Plus) and a diode-array detector (Waters PDA 2996) using an adaptation of the method of Zapata *et al*. [52], with a C8 reverse phase column and a pyridine mobile phase). To determine the fulvic acids content, a modification of the method described by Hautala *et al*. [53] was used: 1) the samples were acidified to pH < 2.5 with 1N HCl; 2) twenty-four hours after the acidification, the samples were filtered through a Whatman GF/C filter to eliminate the precipitates of humic acids; 3) the fulvic acids concentration was obtained through spectrophotometry at 350 nm using a UV-1600PC spectrometer (Model VVVR) and applying the regression described in Gan *et al*. [54]. The macrophyte biomass (g dry weight/m^2^) was estimated as the mean dry weight of three replicates of 50.26 cm^2^ that were taken randomly from each pond. The dry weight was obtained after oven-drying the material at 60 °C over 48 hours.

The maximum surface of the different ponds were estimated using the Google Maps Area Calculator Tool [55] and then checked in the field. Distance to the nearest pond (DNP) was obtained as the straight-line distance between the central point of the studied pond and the central point of the closest pond using Google Maps.

#### Macrofauna and plant sampling

At both sites, the macrofauna (i.e., macroinvertebrates and amphibians) was sampled once in April, at the same time the environmental characteristics were measured, using a dip-net with a diameter of 22 cm and a mesh size of 250 μm. However, since a gradient of pond sizes exists, each sample was obtained from the same sampling effort (20 dip-net sweeps) but from a different number of dip-net sweeps depending on the size (more details in [56]). Samples were preserved *in situ* in ethanol 96%. Subsequently, in the laboratory, the fixative of the samples was removed, and individuals were sorted, counted and identified to species level whenever possible, except in the case of chironomids, which were identified to subfamily level.

Plant species presence was recorded three times during the season (March, April and May) to identify the highest number of species considering their different growing periods. The surveys were carried out by walking throughout each pond from the outer edge to the centre according to the hydrological gradient surveying all different plant communities. Taxa were identified at species and intraspecific levels.

### Data analyses

#### Community similarity and data matrices

We established three biotic groups with different dispersal strategies: macrofaunal active dispersers (AD), macrofaunal passive dispersers (PD) and plants (PL). All analyses were performed separately for each biotic group within each pond network.

We calculated the similarity in community composition between all pond pairs from each network using the Jaccard dissimilarity with presence-absence data (see S1 and S2 Tables), and then, we removed similarities equal to zero to increase the power of the distance-decay approach [30]. We had very few similarities equal to zero, which implies an increase in the explanatory power of the regressions and no problem with dissimilarity saturation [57]. Geographical distances were calculated from the UTM coordinates as Euclidean distances. We used BIO-ENV [58] to identify the subset of environmental variables (previously standardized) among all the variables that we had measured (see subsection ‘*Environmental and geographic parameters’*) from each site that produced the highest correlation with community similarities. Then, we considered the selected variables in each of the networks. This best subset of variables was then used to calculate the environmental distance matrix based on the Euclidean distances between ponds for each pond network. The R package ‘vegan’ was used for the BIO-ENV analyses.

#### Environmental variability assessment

The similarities among the environmental matrices between both pond networks were tested with an analysis of multivariate homogeneity of group dispersions (PERMDISP; [59]). We conducted the analysis based on Euclidean distances of log-transformed environmental variables except for pH and % of fulvic acids. This test was run to guarantee that only the spatial extent and not environmental variation differed among the pond networks. In addition, a general NMDS considering all the environmental variables from the two pond networks and three specific NMDS considering the subset of environmental variables (selected with the BIO-ENV) for each biotic group were carried out to visually assess variations in the distribution of the ponds from each network. The PERMDISP and NMDS analyses were run using PRIMER v.6.

#### Environmental versus geographical distance

First, to determine if there was covariation between the environmental and the geographic distances, we performed Mantel tests (with 1000 permutations) between both distance matrices. The Euclidean distances among the samples (environmental, previously standardized, and geographical coordinates) were calculated, separately for each pond network, to obtain the distance matrices that were then correlated with the Mantel test. Our results indicated that there was no significant correlation in any case (see S4 Table). Second, to assess the influence of environmental distance on community similarity given the geographical distance, and *vice versa*, we performed partial Mantel tests (with 1000 permutations), both ranked and non-ranked (i.e., using Spearman and Pearson correlations, respectively). Since we did not find substantial differences in the results between the ranked and non-ranked tests, and all the significant relations that appeared for the ranked version also appeared for the non-ranked, we only show the results from the non-ranked Mantel tests. These analyses were conducted with the R package ‘ecodist’ [60].

#### Environmental and geographical distance-decay

To analyse the distance-decay patterns (both due to geographical distances and to environmental distances), we used regression models. The similarity between the pairs of samples (response variable) was expressed as (1- Δy) with Δy [0 ≤ Δy ≤ 1] being the change in community structure from one pond (*i* = 1, …, *N*) to another (*j* = 1, …, *N*), as measured by the Jaccard pairwise dissimilarity measure [61]. The Euclidean distance among the samples (environmental, previously standardized, and geographical coordinates) was used as the explanatory variable. We performed three types of regression models according to [15]: linear, exponential, and power-law to know which was the best fit in each case. Since we had better fits with the exponential regression model, we only show the results for this model (the remaining models are included in S5 Table). We tested the significance of the regression models using a randomization procedure with 5000 iterations [62]. Finally, when the regressions for one biotic group were significant in both networks, we tested the difference in slopes using a permutation procedure with 1000 iterations with the ‘diffslope’ function from the ‘simba’ R package [63]. Similarly, we also tested the difference in the slopes among the biotic groups of the same pond network.

## Results

In both pond networks, the pattern of taxa richness among the biotic groups was the same. In the SEPN, the richest group was the PL (74 taxa), followed by the AD (66 taxa) and finally the PD (13 taxa). In the case of the LEPN, the total richness of each group was 102 (PL), 55 (AD) and 27 (PD). With respect to community similarity (Jaccard index), within the SEPN the highest value was 0.833 for the PD, and the lowest value was 0.073 for the PL. Within the LEPN, the AD had the highest value (0.667), and the PD had the lowest value (0.118).

**Fig 2:**
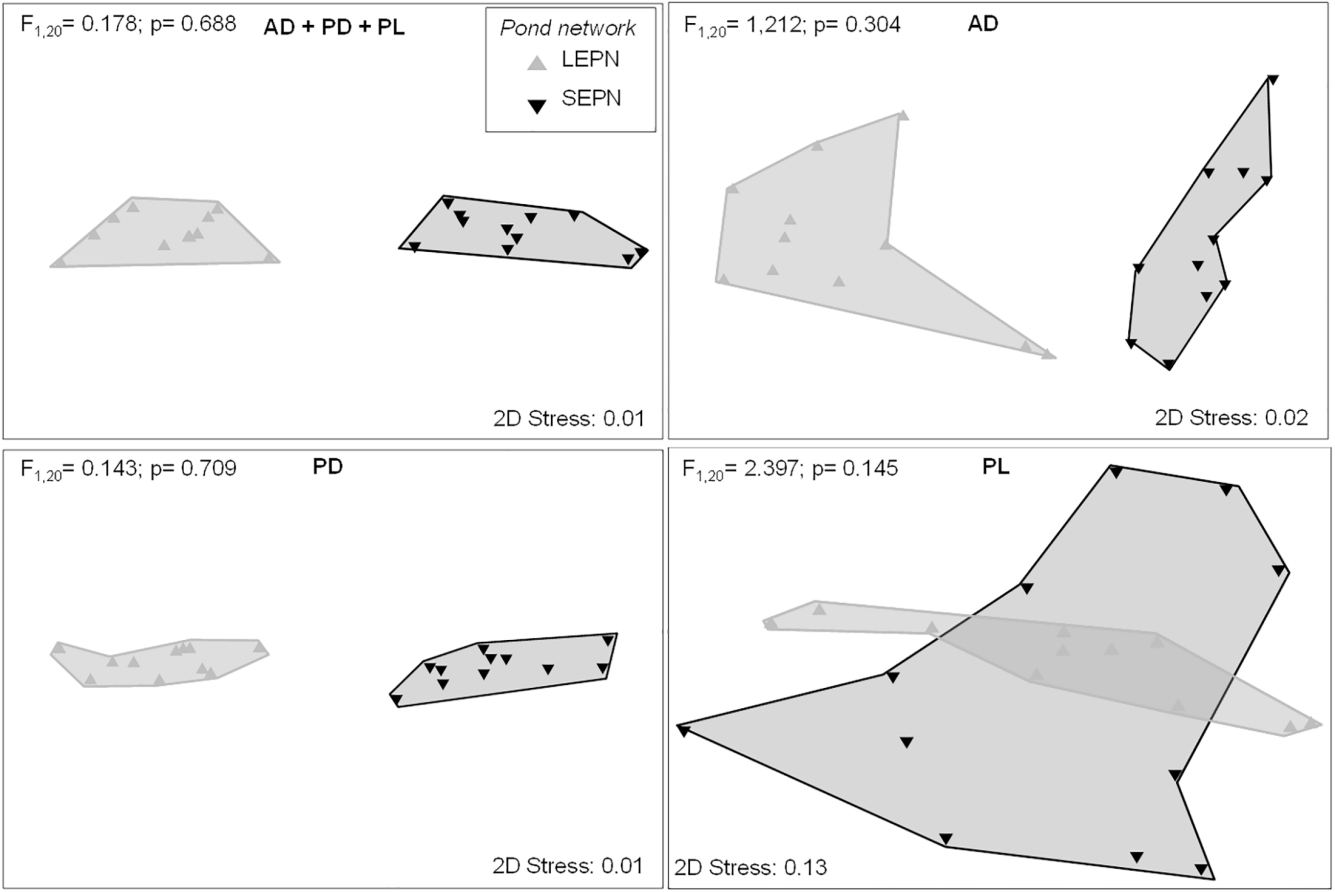
Non-metric multidimensional scaling (NMDS) ordinations of the ponds considering the whole environmental matrix (Euclidean distances) and the three biotic groups (top, left); considering only the environmental variables with the highest correlation for AD (top, right), PD (bottom, left) and PL (bottom, right) (see Table 2). PERMDISP results are shown.

In relation to the measured environmental and geographic parameters, the pond networks have contrasting values of TIC, DIN and % of fulvic acids (S3 Table). However, with the PERMDISP results, we validated that the environmental variability observed in both pond networks is not significantly different (see Fig 2), although the variables that explain the higher proportion of variability are different at each site (Table 1). BIO-ENV analyses identified different sets of environmental variables for each biotic group and pond network, although TOC was selected for almost all the cases (Table 1). The overall correlation with environmental factors was stronger for AD than for PD and PL, independent from the pond network.

**Table 1:** Set of environmental variables identified by the BIO-ENV analysis and the overall correlations (Pearson) for each biotic group and pond network.

Biotic group	Environmental variables for SEPN	Pearson’s R SEPN	Environmental variables for LEPN	Pearson’s R LEPN
AD	TIC, pond size	0.627	max. depth, pH, T, TOC	0.722
PD	Conductivity, pH, T, phosphate, TOC, TP	0.455	TOC, fulvic acids, macrophyte DW, pond size	0.701
PL	oxygen, chorophyll-*a*	0.304	Conductivity, TIC, TOC, pond size, DNP	0.477

Abbreviations are AD (macrofaunal active dispersers), PD (macrofaunal passive dispersers), PL (plants), SEPN (small extent pond network), LEPN (large extent pond network), TIC (total inorganic carbon), T (temperature), TOC (total organic carbon), TP (total phosphorus), DW (dry weight), and DNP (distance to the nearest pond).

According to the partial Mantel tests that we performed to evaluate the relative influence of the environmental and geographical distances on community similarity, we observed that community similarity was generally more strongly related to environmental distance when geographical distance was controlled for, than *vice versa* (Table 2). In fact, geographic distance was not significant in any of the cases. Environmental distance, in contrast, was significant for the AD in both pond networks and for the PD only in the LEPN (Table 3). Moreover, in the LEPN, the correlation between environmental distance and community similarity was higher for the AD (0.681) than for the PD (0.586) (Table 2).

**Table 2:** Partial non-ranked Mantel correlations between community similarity and environmental distance controlling for geographic distance, and *vice versa*, for each biotic group and pond network.

Distance	Pond network	Biotic group	Mantel r
Geographic (controlling for environmental)	SEPN	AD	0.234 (*p =* 0.064*)
		PD	0.172 (*p =* 0.127)
		PL	0.191 (*p =* 0.088*)
	LEPN	AD	-0.063 (*p =* 0.699)
		PD	-0.030 (*p =* 0.610)
		PL	0.140 (*p =* 0.102)
Environmental (controlling for geographic)	SEPN	AD	0.480 (*p =* 0.024**)
		PD	0.304 (*p =* 0.102)
		PL	-0.116 (*p =* 0.657)
	LEPN	AD	0.681 (*p =* 0.001**)
		PD	0.586 (*p =* 0.005**)
		PL	0.340 (*p =* 0.064*)

Statistical significance for each partial Mantel correlation value is given in parentheses. Abbreviations are SEPN (small extent pond network), LEPN (large extent pond network), AD (macrofaunal active dispersers), PD (macrofaunal passive dispersers) and PL (plants).

**Significant differences (p < 0.05).

*Marginally significant differences (0.1 > p <0.05).

**Table 3:** Regression parameters for the relationship between community similarity and distance (geographic and environmental) for each biotic group in the SEPN and LEPN.

Distance	Pond network	Biotic group	R^2^	p-value	Slope
Geographic	SEPN	AD	0.091	0.014**	-1.989*10^−4^
		PD	0.027	0.119	-
		PL	-0.006	0.420	-
	LEPN	AD	-0.017	0.749	-
		PD	-0.019	0.973	-
		PL	-0.010	0.501	-
Environmental	SEPN	AD	0.257	<0.001**	-0.087
		PD	0.089	0.015**	-0.041
		PL	-0.017	0.783	-
	LEPN	AD	0.457	<0.001**	-0.101
		PD	0.320	<0.001**	-0.159
		PL	0.110	0.008**	-0.081

Abbreviations are SEPN (small extent pond network), LEPN (large extent pond network), AD (macrofaunal active dispersers), PD (macrofaunal passive dispersers), and PL (plants).

**Significant differences (p < 0.05).

**Fig 3:**
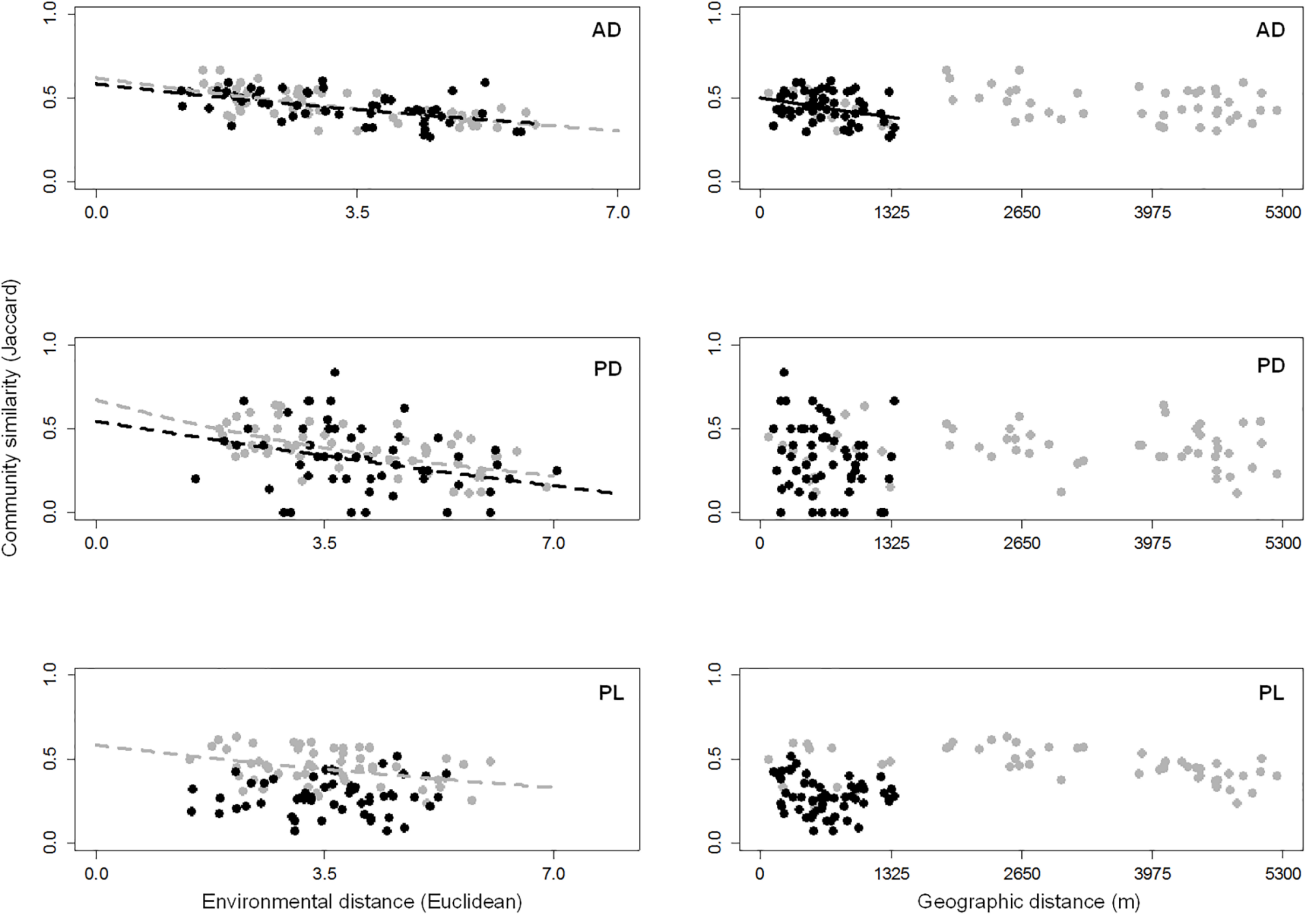
Relationship between community similarity and environmental and geographic distances for AD, PD and PL. Abbreviations are AD (macrofaunal active dispersers), PD (macrofaunal passive dispersers) and PL (plants). The relationship was best approximated by an exponential model in each case (for regression equations see Table 3). Only significant relationships are shown. Black dots and lines are the data from the SEPN (small extent pond network) and grey dots and lines from the LEPN (large extent pond network).

Similar results were obtained when analysing distance-decay patterns. Thus, geographic distance-decay patterns were significant only for the AD in the SEPN (Fig 3). In contrast, environmental distance-decay patterns were detected in all cases, except for the PL in the SEPN. No significant differences were found when comparing the environmental distance-decay slopes of the AD and PD (i.e., comparison between groups) and within pond networks (i.e., comparing the same group between the pond networks), which suggests a general environmental distance-decay response of these two biotic groups regardless the spatial extent of the pond network. Within the LEPN, a significant difference in the slopes of the environmental distance-decay for the PD and PL was the only difference detected (*p* = 0.041) as it was lower than the slope of the PL (difference in slope PD-PL = -0.0781).

## Discussion

In accordance with the expected prevalence of species sorting in aquatic systems [64], we found that all biotic groups showed significant distance-decay relationships with environmental distances suggesting that the metacommunities studied had some environmental control. The slopes were consistent for the faunal active and passive dispersers, regardless of the spatial extent of the site, suggesting that the turnover in species composition due to environmental changes is characteristic of each biotic group (at least for the two faunal groups included in the study). In addition to this environmental control of faunal groups, our findings also confirmed the existence of differences in distance-decay patterns among taxa groups depending on their dispersal strategies and the spatial extent of the pond network [15,65–67]. In this sense, we expected that geographic distance would be more important than environmental distance for the two passively dispersing groups (i.e., PD and PL) due to their likely dispersal limitations [17], but we did not find any significant relationship. The partial Mantel tests indicated that an effect of geographic distance on community similarity was absent. Moreover, the distance-decay relationships were also consistent with this finding, reinforcing the concept that geographic distance plays a very weak role in comparison to environmental distance in these metacommunities. This result could be attributed to an absence of dispersal limitations in the vectors implied in passive dispersal since we observed an absence of dispersal barriers in the two pond networks studied. The lack of a significant relationship with spatial distance for the passive dispersers (PD and PL) reinforces the concept of a strong species sorting effect ruling their metacommunities. In contrast, the AD showed spatial distance-decay but only in the pond network with the smaller spatial extent. This result is consistent with the higher importance of mass effects over other metacommunity dynamics at smaller spatial extents [32], and our results indicate that this mass effects is more evident for the active dispersers than for the passives. When comparing among the groups within the same pond network, there were no significant differences in their slopes except between the PD and PL in the LEPN. Moreover, it is important to note that the existence of different patterns in the same group between pond networks could be related to a difference in the strength of the environmental gradient between the sites [17,64]. However, in our study, we might reject this possibility as both pond networks had similar environmental distances and environmental variability. Therefore, although the length of environmental gradients usually increases with an increasing spatial extent [64], we did not find this effect. Thus, our results are consistent with the concept that different biotic groups with differential dispersal abilities can respond similarly to the same environmental gradients [27].

We confirmed that the organisms with different dispersal modes have different responses to the spatial extent of the pond network [32]. According to previous studies [15,19,30,33], we expected steeper slopes in the SEPN than in the LEPN. However, we were not able to accept or reject this hypothesis since we only found one geographic distance-decay relationship. Our results indicated some role of spatial distance on the active dispersers, and not on the passive taxa (either PD or PL), that was only detected at the smaller spatial extent since the group presented a significant decline in similarity with an increasing spatial distance. It is possible that we did not detect the effect of geographical distance in the LEPN due to the dominance of species sorting (i.e., environmental control) since species sorting appears to be the most important mechanism structuring communities at various spatial extents [64].

However, we are aware that despite the different spatial extents of the studied networks (the LEPN is almost 4 times larger than the SEPN), the maximum extent of the larger pond network is quite small (< 6 km) compared with the distances in other studies [13,66,68]. In fact, most of the studies on the spatial decay of community similarity have considered very large extents (e.g., [10,27,69]), and only a few of the studies focused on areas with small spatial extents (e.g., [33,70,71]). We agree, then, with Steinbauer *et al*. [30] on the concept that more studies at ecosystems with smaller spatial scales are needed to find a more general conclusion. Furthermore, Heino [12] highlighted the more realistic approach of studying metacommunities within small spatial extents than across larger extents since metacommunity dynamics are more likely to act within ecologically defined regions.

Since both pond networks are at similar latitudes and their environmental variability is not significantly different, we might discard these factors as possible explanations for the differences found [15,72,73]. In addition, the fact that environmental and geographic distances were independent makes the results truly reliable when detecting a distance-decay relationship [17,66]. In addition, we have also avoided problems related to comparisons between studies that were differently designed because we followed the same sampling design for the biotic groups in both networks, and we also resolved the ‘sampling effort’ effect despite the pond size gradient [30]. As we already noted, there are few studies that have analysed the distance-decay relationship at small spatial extents. Thus, our study is important since it tries to increase the knowledge on this issue at small spatial extents, and this study highlights the concept that the type of metacommunity dynamics ruling community similarities is strongly influenced by spatial extent [32]. It is highly likely that our results differ from those in studies that performed similar analyses because they were conducted at larger spatial extents. Furthermore, it is important to note that studying two small pond networks ensured that we studied one single metacommunity in contrast with the studies that encompass very large spatial extents [12]. Although similar studies from ponds also consider the species sorting as the main dynamics structuring the communities (e.g., [74]), we can not discard the possibility that the patterns observed and the underlying mechanisms are context dependent (e.g., [75]) since we only studied two pond networks. In line with this, there are regional factors that may differ among sites that we did not consider in our study. These factors are, for instance, the effect of a prevailing wind direction [76] that may, in turn, have a different effect on active and passive dispersers, or the differences among sites regarding the species that may act as vectors of dispersion such as mammals, waterbirds or amphibians (e.g., [77–79]).

Finally, the group of PL from the SEPN was the only group that did not show a significant decay pattern. This result could be explained by the fact that plant communities likely disperse over scales that exceed those in this pond network, and hence, despite the existence of an environmental gradient, the effect of this gradient remains masked by the high exchange between the ponds. In contrast, previous studies did find a negative relationship between similarity and distance for different types of plant communities. However, most of the studies were conducted at very large spatial extents (e.g., [17,80–82]).

In conclusion, our results show that the metacommunity dynamics occurring in each pond network were different and that although mass effects are usually the prevailing mechanism at small spatial extents [32], we found evidences of greater importance from other metacommunity dynamics. Nevertheless, we tried to link one metacommunity dynamics with the patterns found for each biotic group studied, and we were aware that some authors considered this a misconstruction since they encourage future investigations to cover the full spectrum of metacommunity theory [32,72]. Thus, we wanted to highlight the prevalence of one type of metacommunity dynamics over each biotic group but we did not overlook the remaining metacommunity dynamics since it is likely that they are all playing interactive roles [4]. In the case of the LEPN, the three groups studied seem to follow the idea of species sorting perspective indicating that environmental conditions are mainly responsible for structuring these metacommunities independently of organism dispersal modes. This result is consistent with other studies indicating that species sorting prevails in metacommunities over other mechanisms [68,82,83]. Hence, in the LEPN, communities appeared to be homogenized by dispersal to a degree [69], while in the SEPN, each group was likely to be mainly driven by a different mechanism: mass effects for the AD, species sorting for the PD and a pattern that was difficult to associate with any metacommunity mechanism for the PL. Therefore, our results demonstrate the importance of studying metacommunity dynamics and distance-decay at smaller spatial extents since we found differences with respect to studies performed at larger spatial extents and between pond networks that cover a small spatial extent.

## Supporting information

S1 Table Presence/absence data matrix and environmental variables from the SEPN. (XLSX)

S2 Table Presence/absence data matrix and environmental variables from the LEPN. (XLSX)

S3 Table Mean and range of the variation in the environmental and geographic parameters from each pond network. Abbreviations are SEPN (small extent pond network), LEPN (large extent pond network), DIN (dissolved inorganic nitrogen), TIC (total inorganic carbon), DIC (dissolved inorganic carbon), DOC (dissolved organic carbon), TOC (total organic carbon), and DNP (distance to the nearest pond).
(DOCX)

S4 Table Mantel correlations between the environmental distance taking into account only the variables selected by the BIO-ENV analyses for each biotic group and network and the geographic distance. Abbreviations are SEPN (small extent pond network), LEPN (large extent pond network), AD (macrofaunal active dispersers), PD (macrofaunal passive dispersers) and PL (plants).
(DOCX)

S5 Table Regression parameters of the two regression models (linear and power-law) explored for the SEPN and LEPN. Acronyms stand for SEPN (small extent pond network), LEPN (large extent pond network), AD (macrofaunal active dispersers), PD (macrofaunal passive dispersers) and PL (plants).
(DOCX)
