# Supplementary material for: Dispersal mode and spatial extent influence distance-decay patterns in pond metacommunities
Source: PLoS One. 2018 Aug 28;13(8):e0203119. doi: 10.1371/journal.pone.0203119 (PMC6112654; doi:10.1371/journal.pone.0203119)
Supplement: S3 Table — Abbreviations are SEPN (small extent pond network), LEPN (large extent pond network), DIN (dissolved inorganic nitrogen), TIC (total inorganic carbon), DIC (dissolved inorganic carbon), DOC (dissolved organic carbon), TOC (total organic carbon), and DNP (distance to the nearest pond). (DOCX) [file pone.0203119.s003.docx]

| Parameter | Mean (Range) | |
| --- | --- | --- |
|  | SEPN | LEPN |
| Water temperature (°C) | 21.558 | 18.22 |
|  | (17.400-23.900) | (11.800-25.300) |
| Dyssolved oxygen (mg/L) | 4.584 | 10.61 |
|  | (2.817-6.607) | (8.080-13.350) |
| Conductivity (μS/cm) | 778.939 | 420 |
|  | (428.000-1274.667) | (238.000-614.000) |
| pH | 6.543 | 9.474 |
|  | (5.990-7.283) | (7.800-10.550) |
| Maximum depth (cm) | 59.636 | 26.636 |
|  | (30.000-106.000) | (14.000-59.000) |
| Ammonium (mg NH^4+^-N/L) | 0.013 | 0.032 |
|  | (0.001-0.052) | (0.015-0.049) |
| Nitrite (mg NO^2 -^-N/L) | 0.004 | 0.000 |
|  | (0.003-0.006) | (0.000-0.000) |
| Nitrate (mg NO^3-^ -N/L) | 0.003 | 0.007 |
|  | (0.002-0.009) | (0.000-0.067) |
| Phosphate (mg PO^3−^_4_ -P/L) | 0.017 | 0.001 |
|  | (0.004-0.096) | (0.001-0.003) |
| DIN (mg N/L) | 0.02 | 18.147 |
|  | (0.006-0.066) | (14.525-20.851) |
| TIC (mg C/L) | 15.276 | 8.672 |
|  | (5.231-75.190) | (4.431-11.430) |
| DIC (mg C/L) | 13.873 | 7.386 |
|  | (2.058-73.640) | (3.796-11.130) |
| DOC (mg C/L) | 54.675 | 11.787 |
|  | (40.140-79.440) | (7.619-19.980) |
| TOC (mg C/L) | 57.779 | 12.548 |
|  | (40.140-80.120) | (8.000-20.730) |
| Total Nitrogen (mg NT-N/L) | 2.764 | 1.137 |
|  | (1.874-3.798) | (0.695-1.726) |
| Total Phosphorus (mg PT-P/L) | 0.132 | 0.095 |
|  | (0.034-0.640) | (0.039-0.305) |
| Chlorophyll *a* (μg/L) | 11.224 | 4.878 |
|  | (0.271-40.868) | (1.199-14.424) |
| % Fulvic acids | 63.085 | 21.504 |
|  | (50.889-77.487) | (8.858-33.339) |
| Macrophyte biomass (g DW/cm^2^) | 0.194 | 0.008 |
|  | (0.120-0.313) | (0.004-0.015) |
| Pond surface (m^2^) | 18637.727 | 20171.545 |
|  | (245.000-78652.000) | (565.000-79990.000) |
| DNP (m) | 179.565 | 305.5 |
|  | (130.450-214.650) | (82.130-474.600) |
