# Supplementary material for: Dispersal mode and spatial extent influence distance-decay patterns in pond metacommunities
Source: PLoS One. 2018 Aug 28;13(8):e0203119. doi: 10.1371/journal.pone.0203119 (PMC6112654; doi:10.1371/journal.pone.0203119)
Supplement: S4 Table — Abbreviations are SEPN (small extent pond network), LEPN (large extent pond network), AD (macrofaunal active dispersers), PD (macrofaunal passive dispersers) and PL (plants). (DOCX) [file pone.0203119.s004.docx]

| Pond network | Biotic group | R^2^ | p-value |
| --- | --- | --- | --- |
| SEPN | AD | 0.208 | 0.077 |
|  | PD | 0.188 | 0.112 |
|  | PL | 0.144 | 0.162 |
| LEPN | AD | 0.138 | 0.109 |
|  | PD | 0.059 | 0.241 |
|  | PL | 0.083 | 0.251 |
