# Supplementary material for: Dispersal mode and spatial extent influence distance-decay patterns in pond metacommunities
Source: PLoS One. 2018 Aug 28;13(8):e0203119. doi: 10.1371/journal.pone.0203119 (PMC6112654; doi:10.1371/journal.pone.0203119)
Supplement: S5 Table — Acronyms stand for SEPN (small extent pond network), LEPN (large extent pond network), AD (macrofaunal active dispersers), PD (macrofaunal passive dispersers) and PL (plants). (DOCX) [file pone.0203119.s005.docx]

| Distance | Pond network | Group | R^2^  Linear | R^2^  power-law |
| --- | --- | --- | --- | --- |
| Geographic | SEPN | AD | 0.075 | 0.034 |
|  |  | PD | 0.031 | 0.026 |
|  |  | PL | 0.013 | 0.028 |
|  | LEPN | AD | -0.017 | -0.018 |
|  |  | PD | -0.019 | -0.017 |
|  |  | PL | 0.007 | -0.019 |
| Environmental | SEPN | AD | 0.247 | 0.232 |
|  |  | PD | 0.094 | 0.070 |
|  |  | PL | -0.011 | -0.018 |
|  | LEPN | AD | 0.453 | 0.470 |
|  |  | PD | 0.331 | 0.296 |
|  |  | PL | 0.105 | 0.101 |
